# Case Report: Use of endobronchial Watanabe spigot and coagulation factor XIII supplementation in the treatment of persistent pneumothorax due to pneumocystis pneumonia with human immunodeficiency virus infection

**DOI:** 10.3389/fmed.2022.956333

**Published:** 2022-10-05

**Authors:** Katsumasa Koyama, Daisuke Minami, Hayato Isobe, Ryo Shirai, Niro Okimoto, Koichi Tomoda

**Affiliations:** ^1^Department of General Internal Medicine 1, Kawasaki Medical School, Okayama, Japan; ^2^Department of Respiratory Medicine, Hosoya Hospital, Okayama, Japan

**Keywords:** endobronchial Watanabe spigot, blood coagulation factor XIII, pneumothorax, *pneumocystis jiroveceii* pneumonia, human immunodeficiency virus

## Abstract

*Pneumocystis jiroveceii* pneumonia is one of the most common opportunistic infections associated with human immunodeficiency virus. Endobronchial Watanabe spigot has been recommended for refractory pneumothorax, even with persistant air leak despite continuous negative pressure control *via* thoracic drainage. Moreover, coagulation factor XIII is considered effective in wound healing.

## Introduction

*Pneumocystis jiroveceii* pneumonia (PJP) is one of the most common opportunistic infections associated with human immunodeficiency virus (HIV). A high incidence of pneumothorax that may become intractable has been reported in patients with PJP with HIV infection (1). The use of endobronchial Watanabe spigot (EWS) has been proposed for refractory pneumothorax. However, air leak might persist despite continuous negative pressure control by thoracic drainage. In addition, blood coagulation factor XIII (FXIII) has been reported to be effective in promoting wound healing. Herein, we report a case of refractory pneumothorax secondary to HIV-associated PJP that responded to the use of EWS and FXIII supplementation.

## Case description

A 37-year-old man visited a neighborhood hospital with a chief complaint of fever that had persisted for 3 weeks. The patient had no history of smoking or alcoholic drinking. Furthermore, his medical history was generally unremarkable. He works as a care staff and denied having a history of exposure to dust. Chest X-ray revealed pneumonia, prompting referral to our hospital.

**Figure 1:**
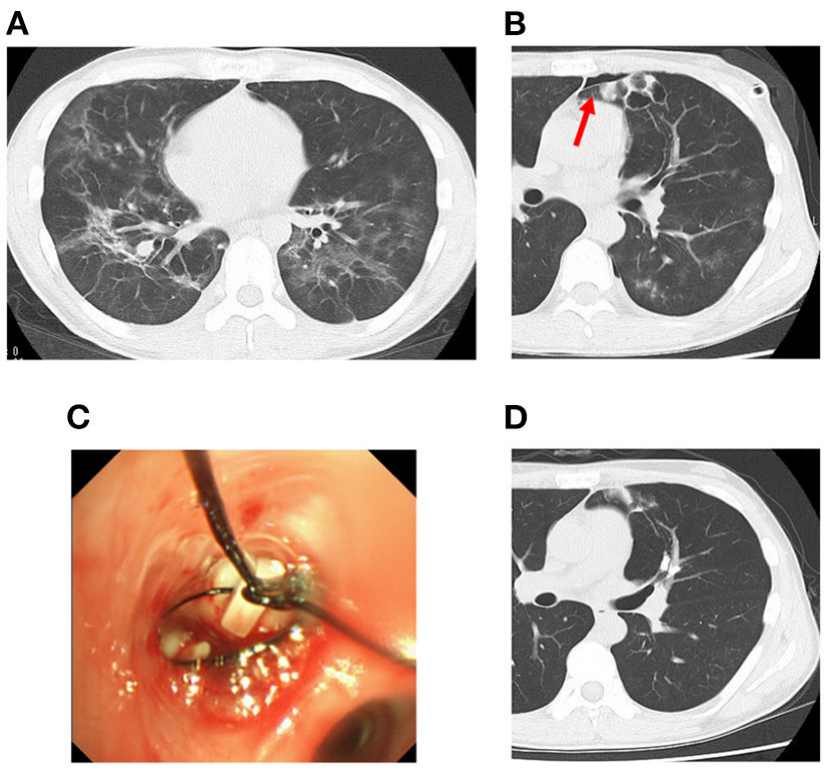
Chest computed tomography (CT) scan on admission **(A)**, at 11 days **(B)**, and after bronchial occlusion with a endobronchial Watanabe spigot (EWS) **(D)**, and EWS **(C)**. CT scan of the chest showing diffuse ground glass opacity on admission **(A)**. CT scan of the chest showing cysts clustered on the peripheral side of the left B4 at 11 days **(B)**. EWS shown being implanted in left B4b **(C)**. And, CT scan showing improvement in the pneumothorax after bronchial occlusion with EWS **(D)**.

Computed tomography (CT) scan showed diffuse ground glass opacities in both lungs (Figure 1A). He was admitted to our hospital for further diagnosis and treatment. On physical examination on admission, his height and body weight were 167.1 cm and 54.7 kg, respectively. The following vital signs were checked: body temperature, 37.3°C; blood pressure, 108/50 mmHg; and pulse rate, 66 beats per min. The patient was fully active, with an Eastern Cooperative Oncology Group performance status score of zero. The level of transcutaneous arterial oxygen saturation was 98% on room air. The bulbar conjunctiva did not exhibit significant pallor indicative of anemia. Cardiac examination revealed no abnormalities. In addition, there were no adventitious lung sounds. Abdominal and neurological findings were also normal. The patient presented with redness on the entire face. The skin of the hands was rough and covered with scales. Additionally, depression in the fingernails and toenails was observed. Interdigital scales were also noted on the left foot. Laboratory test results on admission (Table 1) showed elevated inflammatory markers: white blood cell (WBC), 4,070/μl (neutrophil, 68.0%) and C-reactive protein (CRP), 3.38 mg/dl. Liver and renal function tests were almost within normal range. The patient tested positive for the HIV antigen and his CD4 count was markedly reduced (27/μl). Transbronchial lung biopsy, bronchial brushing, and bronchoalveolar lavage were performed from the left B8a. Histological and cytological analysis (Grocott's staining) detected *Pneumocystis jirovecii* in the foamy substance from the pathological specimens. None of the findings were suggestive of malignancy or presence of cytomegalovirus.

**Table 1:** Laboratory findings.

**Variable**	**Result**	**Reference range**	**Variable**	**Result**	**Reference range**
	**Hematology**			**Biochemistry**	
Red blood cells (× 10^4^/μl)	407	435–555	Sodium (mmol/L)	134	138–145
Hemoglobin (g/dl)	13.0	13.7–16.8	Potassium (mmol/L)	4.0	3.6–4.8
Hematocrit (%)	39.0	40.7–50.1	Chlorine (mmol/L)	107	101–108
White blood cells (/μl)	4,070	3,300–8,600	BUN (mg/dl)	9	8–20
Nt. (%)	68.0	40.7–50.1	Creatinine (mg/dl)	0.63	0.65–1.07
Lym. (%)	21.1	15.8–34.8	Total bilirubin (mg/dl)	0.30	0.4–1.5
Eos. (%)	1.0	1.0–5.0	AST (U/L)	25	13–30
Bas. (%)	0.0	6.6–8.1	ALT (U/L)	6	10–42
Mon. (%)	9.9	4.1–5.1	LDH (U/L)	357	124–222
Mon. (%)	9.9	4.1–5.1	Albumin (g/dl)	1.9	4.1–5.1
Platelets (× 10^4^/μl)	14.3	15.8–34.8	Total protein (g/dl)	5.6	6.6–8.1
	**Collagen disease-related antibodies**		CK	18	59–248
Anti-nuclear Ab. (IU/L)	< 10	0–12	CRP (mg/dl)	3.38	34.0–46.0
Rheumatoid factor (IU/L)	< 15	0–15	KL-6 (U/ml)	460	< 500
Anti-CCP Ab. (IU/L)	< 0.5	< 4.5	CD4	3.7	
Anti-ds-DNA Ab. (IU/L)	< 10	0–112	CD8	75.2	
Anti-RNP Ab. (IU/L)	< 2.0	< 10.0	CD4/8	0.05	
Anti-Scl-70 Ab. (IU/L)	< 1.0	< 10.0	C7-HARP	–	–
Anti-SS-A Ab. (IU/L)	< 1.0	< 10.0	β-D	108.8	0.0–20.0
Anti-ARS Ab. (IU/L)	< 5.0	< 25.0	QFT	–	–
PR3-ANCA	< 1.0	< 3.5	HIV-Ab	+	–
MPO-ANCA	< 1.0	< 3.5			

ALT, alanine aminotransferase; AST, aspartate aminotransferase; Bas, basophils; BUN, blood urea nitrogen; CRP, c-reactive protein; Eos, eosinophils, Hb, hemoglobin; Hct, hematocrit; HIV-Ab; human immunodeficiency virus antibodies; KL-6, Krebs von den Lungen-6; LDH, lactate dehydrogenase; Lym, lymphocytes; Mon, monocytes; RBC; Nt, neutrophils; PLT, platelets; QFT, QuantiFERON^®^-TB Gold; RBC, red blood cells; T-Bil, total bilirubin, WBC, white blood cells; β-D, beta-D-glucan.

A detailed interview aimed to identify the specific type of pneumonia revealed a history of homosexual intercourse during a 6-month period 13 years before admission. After providing informed consent, the patient tested positive for HIV. Based on this result and bronchoscopy findings, he was diagnosed with PJP complicated by HIV infection. Therefore, treatment with sulfamethoxazole-trimethoprim (nine tablets/day for 23 days) was started. Poor oxygenation was observed during the treatment course, with a level of transcutaneous arterial oxygen saturation on room air below 90%, prompting addition of prednisolone (40 mg/day for 5 days) to the treatment. The pneumonia tended to improve. However, on day 11 after disease onset, the patient reported left-sided chest pain. Repeat chest X-ray revealed grade III pneumothorax in the left lung. A single chest tube was placed and maintained under continuous negative aspiration pressure for a week. However, air leak persisted, and pleurodesis with a 50% glucose solution was not successful. CT scan of the chest showed cysts clustered on the peripheral side of the left B4 (Figure 1B). This site was identified to be the location of air leak, and bronchial occlusion with an EWS (size: M, 6 mm; Novatech, La Ciotat, France) was performed (Figure 1C). However, air leak decreased after the procedure. However, FXIII activity was reduced to 41% possibly due to the HIV infection. Therefore, intravenous infusion of FXIII (Fibrogammin^®^ 240 IU, 4 ml/day for 5 days) was added to the treatment, enabling drain removal (Figure 1D). The patient subsequently received antiretroviral drug therapy for HIV and was discharged on day 33 after disease onset.

## Discussion

It has been reported that patients with HIV complicated by PJP often had symptoms similar to those of common cold, which precede disease onset by a few weeks and gradually exacerbate. A high incidence (5–10%) of pneumothorax complications have been reported in patients with PJP with HIV (1). In addition, 10–35% of these patients present with cystic changes in the lungs (2). Pneumothorax in these patients requires long-term drainage due to high recurrence and persistence of air leaks. This may be due to pleural necrosis caused by PJP as well necrosis spread to the pulmonary parenchyma (3). In some cases in which pneumothorax does not respond to conservative treatments, such as chest drainage and pleurodesis, surgery is performed. However, surgery is not recommended in patients positive for HIV due to delayed wound healing, possibility of postoperative complications, and potential postoperative worsening of HIV infection. In addition, elevated postoperative mortality has been reported in patients with a low CD4 count (4). Surgery was therefore contraindicated in this particular patient due to the markedly low number of CD4^+^ T cells (27/μl) (5).

Endobronchial spigot placement is an endoscopic treatment developed by Watanabe et al. (6) with the aim of improving various conditions by bronchial occlusion through the endoscopic occlusion of segmental and subsegmental bronchi. Secondary pneumothorax without adequate lung expansion due to persistent air leak, pulmonary fistula, and empyema with bronchopleural fistula are indications for EWS placement (7). EWS placement is also appropriate in patients at high risk for surgery, such as the one described in this report. We selected and performed EWS due to possible risk of pneumonia exacerbation after valve placement in the tracheal tube. Since EWS is simple and minimally invasive treatment, it was an appropriate option in our patient. In addition, valve placement was not covered by insurance in Japan. Therefore, we could not consider valve placement according to the general clinical situation. On the other hand, since valve placement was difficult to preform, EWS, which has a high safety profile and made of medical silicone, could be often implanted for a long period of time (8). In our case, cysts that clustered on the peripheral side of the left B4 disappeared and secondary pneumothorax was improved after EWS for about 1 year. In addition, his condition regarding CPC stabilized. Therefore we considered to remove the spigots.

FXIII deficiency has been associated with failure of wound healing and fistula (9). Unlike direct occlusion of fistula, as is the case in surgery and pleurodesis, FXIII supplementation aims to promote spontaneous patient recovery. The mechanism of action of FXIII involves enhancement of wound healing through promoting fibroblast proliferation, bridging of the gap using fibrin scaffolds, and making changes into the favored net structure. FXIII supplementation is indicated for cases of anastomotic failure and fistula in which FXIII activity is 70% or less than the normal values. FXIII supplementation has been reported to be effective in fistula and anastomotic failure after gastrointestinal surgery, as well as in 70% of patients with pulmonary fistula that persisted at least 5 days after lung lobectomy. It has also been reported to be useful after surgery for secondary pneumothorax caused by pneumocystis pneumonia. In Japan, the local application of FXIII supplementation is not covered by insurance. Howeveer, the efficacy of FXIII systemic infusion has been reported (10). Therefore, in the current case, FXIII supplementation was reduced to 41% of its normal value. Consequently, intravenous administration of FXIII might have contributed to the observed improvement in the bronchial fistula.

## Conclusion

Herein, we report a case of intractable secondary pneumothorax that originated as a complication of a pneumocystis pneumonia with HIV infection. This case well responded to bronchial occlusion with EWS and treatment with FXIII.

## Data availability statement

The original contributions presented in the study are included in the article/supplementary material, further inquiries can be directed to the corresponding author.

## Ethics statement

Written informed consent was obtained from the individual(s) for the publication of any potentially identifiable images or data included in this article.

## Author contributions

KK researched data and wrote the manuscript. DM, HI, and RS researched data and contributed to the discussion. NO and KT reviewed the manuscript. All authors contributed to the article and approved the submitted version.

## Conflict of interest

The authors declare that the research was conducted in the absence of any commercial or financial relationships that could be construed as a potential conflict of interest.

## Publisher's note

All claims expressed in this article are solely those of the authors and do not necessarily represent those of their affiliated organizations, or those of the publisher, the editors and the reviewers. Any product that may be evaluated in this article, or claim that may be made by its manufacturer, is not guaranteed or endorsed by the publisher.
